# Sex Differences in the Systemic and Local Immune Response of Pancreatic Cancer Patients

**DOI:** 10.3390/cancers15061815

**Published:** 2023-03-17

**Authors:** Azaz Ahmed, Sophia Köhler, Rosa Klotz, Nathalia Giese, Thilo Hackert, Christoph Springfeld, Dirk Jäger, Niels Halama

**Affiliations:** 1 Medical Oncology and Internal Medicine VI, National Center for Tumor Diseases (NCT), University Hospital Heidelberg, University Heidelberg, 69120 Heidelberg, Germany; 2 Translational Immunotherapy, German Cancer Research Center (DKFZ), 69120 Heidelberg, Germany; 3 General, Visceral and Transplantation Surgery, University Hospital Heidelberg, University Heidelberg, 69120 Heidelberg, Germany; 4 Applied Tumor Immunity Clinical Cooperation Unit, National Center for Tumor Diseases (NCT), German Cancer Research Center (DKFZ), 69120 Heidelberg, Germany

**Keywords:** pancreatic ductal adenocarcinoma, pancreatic cancer, sex difference, tumor immunology, CXCL12

## Abstract

**Simple Summary:**

Sex is a factor that affects how the immune system works in both healthy and sick people. There are differences between females and males in various aspects of cancer, such as frequency of diagnosis, treatment response and survival. However, females are often underrepresented in clinical trials that investigate immunological cancer treatments. Further, sex-related differences are rarely studied. Not much is known about how sex affects pancreatic cancer, especially when it comes to the immune system. We looked at cancer tissues and blood samples from pancreatic cancer patients and found that females had a stronger systemic immune response and differential immune responses in the tissue compared to males. We also found that the effects of a specific cancer treatment with the blockade of CXCL12 differed between sexes. These findings show that it is important to consider sex differences when studying pancreatic cancer and immunological treatments.

**Abstract:**

Background: Mounting evidence suggests that sex plays a critical role in various aspects of cancer such as immune responses. However, a male bias exists in human and non-human studies including immunotherapy trials. The role of sex on immune responses in pancreatic ductal adenocarcinoma (PDA) is unclear. Methods: Here, tumor tissues (tumor and stroma separately) and corresponding blood samples from patients with PDA (*n* = 52) were systematically analyzed by immunohistochemistry and multiplex cytokine measurements and compared by sex. Results: Females showed a stronger systemic immune response with higher levels of CXCL9, IL1B, IL6, IL10 and IL13. Additionally, more peripheral white blood cells were detected in females. In the microenvironment, males showed higher tumoral levels of CXCL12. No differences were detected in the stroma. Females showed a tendency towards an anti-tumoral immune cell profile. CXCL12 blockade indicated a differential microenvironmental effect by sex in an independent immunotherapy trial cohort of patients with PDA (one female, five males). The overall survival did not differ by sex in our cohort. Conclusion: Systemic and local immune responses differ between sexes in PDA. Accordingly, sex-dependent differences need to be considered in human studies and for specific immunological interventions before clinical translation.

## 1. Introduction

Sex differences are present in many aspects of cancer, including its incidence, treatment response and clinical outcomes [1,2,3,4]. Moreover, sex is a known biological variable that affects innate and adaptive immune responses, and in the past, sex-associated differences in the efficacy of immunotherapeutic approaches such as immune checkpoint blockade have been reported [5,6,7,8]. Hormone differences (e.g., estrogens, progesterone, androgens) and X and Y chromosome-linked immune genes contribute to regulation differences of immune responses between males and females [7,9]. However, females are underrepresented in immunotherapy trials, and most studies fail to analyze results by sex (only less than 10%) [5]. The male bias is even higher in non-human studies [5]. It is often assumed that results from studies conducted on males can be applied to females. Additionally, the concern that hormonal cycles in females may decrease the homogeneity of study cohorts and potentially confound the effects of experimental procedures is a common reason for the underrepresentation of females in biomedical research. In this discussion, it has even been argued that the scientific community and researchers themselves should take into account when sex is an expected source of variation, indicating no need for specific mandates by health institutions [5,10,11]. These factors contribute to the limited knowledge of female biology in various diseases. In addition to biological factors, gender-related factors also explain clinical differences in male and female patients. These include individual exposure to risk factors such as tobacco, alcohol consumption and obesity [12,13,14].

The understanding of the role of sex in pancreatic ductal adenocarcinoma (PDA) is limited. PDA has a higher incidence in males than females, and the data regarding survival outcomes is controversial, with some studies showing no sex difference and others showing higher mortality in male patients [1,3,12,15]. These clinical differences are not fully explained by patient or treatment characteristics, indicating that biological factors such as immune responses may play a role in PDA and need to be further investigated [12,16].

To comprehensively assess sex-associated immune responses, it is important to consider both systemic and local immune responses, especially in PDA as the typical desmoplastic stroma is a major barrier and one of the main reasons for the “cold” immune nature (low infiltration by antitumor immune cells) of the pancreatic tumor microenvironment (TME).

In this study, we analyzed matched systemic and local immune parameters of human patients with PDA by evaluating cytokine concentrations on a protein level and using serial immunohistochemistry. Additionally, we analyzed patient outcomes between males and females. Our aim was to investigate sex differences in the immune response of patients with PDA, which may potentially improve sex-specific patient management and help tailor personalized (immune) therapeutic strategies.

## 2. Materials and Methods

### 2.1. Patient Cohort

The Department for General, Visceral and Transplantation Surgery at the University Hospital Heidelberg searched its prospectively maintained electronic pancreas database for patients who underwent pancreatic surgery between 03/2007 and 07/2011. Previously untreated patients with PDA with preoperatively obtained serum samples, available frozen tumor tissue samples and available clinical baseline and outcome parameters were included. The local ethics committee of the University of Heidelberg approved tissue and data collection (323/2004). All patients provided written informed consent. To assess local cytokine changes upon CXCL12 blockade, we investigated all patients with PDA (*n* = 9) in the OPERA trial cohort [17]. In the OPERA trial, patients with advanced metastatic PDA were treated with anti-CXCL12 (NOX-A12) for 2 weeks, followed by a combination therapy with pembrolizumab. From the cohort of 9 patients with PDA, serial biopsies (day 0 and day 14) to assess microenvironmental cytokine concentrations before and after anti-CXCL12 treatment were available for 6 patients (one female, five males). Blood samples from healthy controls were provided by the blood bank of the University Hospital Heidelberg and used for reference.

### 2.2. Data Collection and Outcome Parameters

Baseline data were extracted from the database and included the following: sex, age, weight, body mass index (BMI) and comorbidities (cardiovascular, pulmonary, renal, hepatic, autoimmune, diabetes). Preoperative serum values for white blood cells (WBCs) and c-reactive protein (CRP) were obtained from the hospital’s laboratory information system. The overall survival time was also assessed. Pathological reports included pTNM tumor stage according to the TNM Staging Manual, American Joint Committee on Cancer (AJCC), tumor grading and resection margin status [18].

### 2.3. Immunohistochemical Staining

Briefly, 4 μm tissue slices were prepared from cryopreserved tissues and mounted on glass slides. Immunohistochemistry was performed according to the manufacturer’s instructions using an automated staining facility (Bond Max, Leica, Wetzlar, Germany). The following mouse monoclonal antibodies were utilized: CD3ϵ (clone ab16669, 1:100, Abcam, Cambridge, UK; RRID: AB_443425), CD4 (clone 4B12, 1:150, Leica, Wetzlar, Germany; RRID: AB_563559), CD8 (clone 4B11, 1:100, Leica, Wetzlar, Germany; RRID: AB_442068), CD20 (clone L26, 1:100, Leica, Wetzlar, Germany; RRID: AB_442055), CD163 (clone EPR19518, 1:500, Abcam, Cambridge, UK; RRID: AB_2753196), NKp46 (clone 195314, 1:175, R&D Systems, Minneapolis, MN, USA; RRID: AB_2149153) and FoxP3 (clone 236A/E7, 1:100, Thermo Fisher Scientific, Waltham, MA, USA; RRID: AB_467555).

### 2.4. Immune Cell Quantification

Immune cells were quantified using a software-based image analysis system. Whole-slide images were acquired with NanoZoomer 2.0 HT scan system (Hamamatsu, Hamamatsu City, Japan) and scanned at 40× magnification. The cell density in immune cell conglomerates was estimated as previously described [19]. Additional analysis was conducted using image analysis software (VIS software suite, Visiopharm, Hoersholm, Denmark). Quantification algorithms were applied individually for each distinctive staining protocol according to the strength of DAB staining.

### 2.5. Laser Capture Microdissection

This procedure allows the isolation of selected cell populations from a section of complex tissue under direct microscopic observation. Using PDA sections, tumoral and stromal compartments were isolated from each other by laser capture microdissection. This was necessary for separately assessing local cytokine concentrations in the different compartments. The procedure was performed according to the standard protocol of the inventors. Briefly, after focusing on the tissue section (20× magnification), the tumoral and stromal area was manually separated using the Leica Laser Microdissection software (Leica, Wetzlar, Germany). The final dissection was performed with a carbon dioxide laser pulse.

### 2.6. Protein Quantification in Stromal and Tumor Epithelial Compartment

Cryopreserved PDA tissue samples were cut into multiple consecutive sections (20 µm) and then stained with cresyl-violet. Areas of tumor epithelium and stroma were highlighted using brightfield microscopy at 20× magnification. Laser microdissection was performed with the Leica Laser Microdissection V5.0.2.0 software. Sufficient protein concentrations for multiplex analysis could be obtained by the dissection of 30–50 × 106 mm^2^. The tissue was lysed using the BioPlex™ Cell Lysis Kit (Bio-Plex Cell Lysis Kit, BioRad, Hercules, CA, USA; 171304011) according to the manufacturer´s instructions. Serum samples were thawed overnight at 4 °C and then diluted at 1:1 using Sample Diluent (BioRad, Hercules, CA, USA) prior to protein quantification. A two-laser array reader simultaneously quantified all proteins of interest. The concentrations were calculated using Bio-Plex Manager 4.1.1 and a 5-parameter logistic plot regression formula. The Bio-Plex Pro Human Cytokine Screening Panel 48-plex (BioRad, Hercules, CA, USA; 12007283), Bio-Plex Pro Human Cytokine ICAM-1 (BioRad, Hercules, CA, USA; 171B6009M) and Bio-Plex Pro Human Cytokine VCAM-1 (BioRad, Hercules, CA, USA; 171B6022M) were used for the quantification of immunological parameters.

### 2.7. Measurement of White Blood Cells (WBC) and c-Reactive Protein (CRP)

The counts of WBC and CRP concentration (normal range < 5 mg/dL) were determined in blood samples of the PDA patient cohort in the Core Laboratory Facilities of the University Hospital Heidelberg in accordance with accredited standards (DIN EN ISO 15189, D-ML-13060-01-00).

### 2.8. Statistical Analysis

Mann–Whitney tests were used for the comparison of non-paired samples. The latest information was used to assess the overall survival time. Survival curves were visualized using Kaplan–Meier estimators. Overall survival between patient groups was compared using the log-rank test. Significant differences were represented as follows: * *p* ≤ 0.05, ** *p* ≤ 0.005 and *** *p* ≤ 0.0001. All analyses were performed using the statistical software GraphPad Prism 8.4.1. The findings are generally regarded as reportable, even when no statistical significance is observed. Indicators of significance are supplied according to current recommendations [20,21].

## 3. Results

### 3.1. Characteristics of Patients by Sex

The investigated patient cohort consisted of 52 patients in total. Serum samples were obtained prior to surgery and tissue specimens were collected upon surgical resection. All patients were treatment-naïve and had not received prior local or systemic therapy. As described in Table 1, the cohort was equally distributed by sex, with 26 males and 26 females. Males had a higher mean body weight than females (75.4 kg vs. 66.6 kg, *p* = 0.019), but a similar body mass index (BMI) (23.9 kg/m^2^ vs. 25.2 kg/m^2^, *p* = 0.557). The preexistent comorbidities were comparable in both groups, with a higher rate of cardiac comorbidities in females (*n* = 17 vs. *n* = 10, *p* = 0.053). The histopathological workup showed similar results in both groups, and the diagnosis of PDA was confirmed in all samples.

### 3.2. Female Pancreatic Cancer Patients Show a Stronger Systemic Immune Response

**Figure 1:**
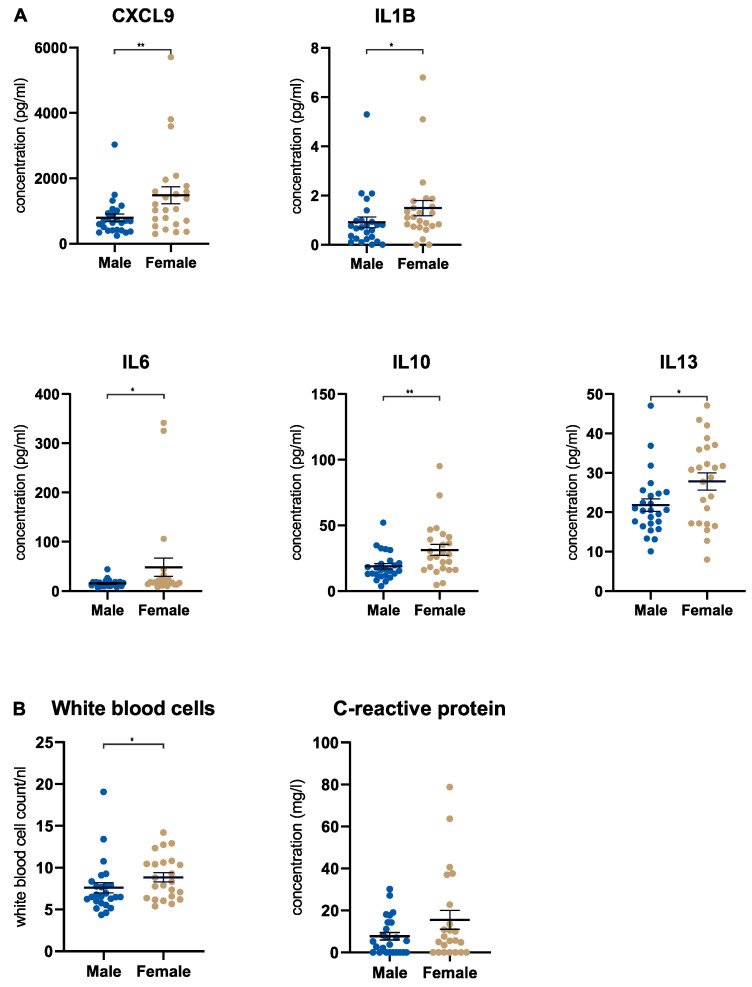
Female pancreatic cancer patients show a stronger systemic immune response. (**A**) Scatter dot plots comparing the serum concentration of immunological parameters (as indicated) in male (*n* = 25) and female (*n* = 24) patients with PDA. (**B**) Scatter dot plots comparing the serum concentration of WBC and CRP in male (*n* = 25) and female (*n* = 23) patients with PDA. PDA = pancreatic ductal adenocarcinoma. WBC = white blood cells. CRP = C-reactive protein. * *p* ≤ 0.05, ** *p* ≤ 0.005.

First, our aim was to investigate systemic immune responses in male and female treatment-naïve patients with PDA by comparing the serum concentrations of 50 soluble factors, including cytokines, chemokines, and growth factors. This analysis showed that female patients displayed a significant upregulation of five systemic factors: CXCL9, IL1B, IL6, IL10 and IL13 (Figure 1A). No other soluble factor showed a significant difference between males and females (Supplemental Figure S1A). Focusing on female patients, we identified a group of patients with high IL13 levels (*n* = 12 of a total of *n* = 26 female patients) whose IL13 levels were above the mean ± SEM. Examining the clinical characteristics of these females with high IL13 levels, some characteristics differentiated the IL13 high group from the IL13 low group: the IL13 high group had a higher mean age (69.9 years vs. 63.6 years) and more patients with diabetes mellitus (42% vs. 21%) and cardiac comorbidities (83% vs. 57%) (Table S1). Additionally, we analyzed routine inflammation markers in all patients. The count of white blood cells (WBCs) in the serum was significantly higher in females, whereas the levels of c-reactive protein (CRP) did not differ significantly (Figure 1B). As expected, serum IL6 and CRP showed a positive correlation (r = 0.49; *p* = 0.0007) in the full cohort (Supplemental Figure S1B). To assess if the alterations in the serum levels of CXCL9, IL1B, IL6, IL10 and IL13 were associated with PDA, we compared male and female patients with PDA to a cohort of healthy individuals (*n* = 30). Our analysis revealed that changes in these cytokines were cancer-related. Specifically, we observed a significant downregulation of CXCL9 and IL1B and a significant upregulation of IL6, IL10 and IL13 in both male and female patients with PDA compared to the healthy control group (Supplemental Figure S1C).

### 3.3. Male Patients Express Higher Tumoral Levels of CXCL12

**Figure 2:**
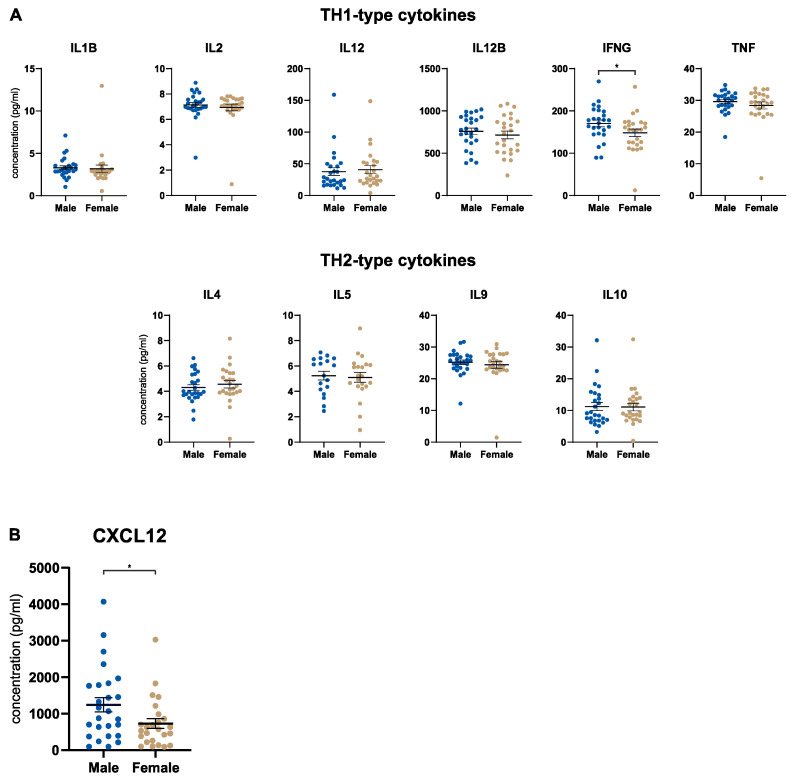
Male patients express higher tumoral levels of CXCL12. (**A**) Scatter dot plots comparing the tumoral concentration of immunological parameters (as indicated) in male (*n* = 26) and female (*n* = 26) patients with PDA. The parameters were categorized into TH1- and TH2-type cytokines. (**B**) Scatter dot plot comparing the tumoral CXCL12 concentration in male (*n* = 26) and female (*n* = 25) patients with PDA. PDA = pancreatic ductal adenocarcinoma. * *p* ≤ 0.05.

The next step was to determine the concentration of the same 50 factors in the pancreatic tumor microenvironment (TME). Of note, we focused on the tumoral and stromal compartments separately after performing laser capture microdissection on the resected tissues. In the tumor tissue, we investigated cytokine signatures related to TH1 or TH2 responses and did not detect an overriding sex-specific immune pattern in this regard (Figure 2A). Although the concentration of IFNG was significantly upregulated in male patients with PDA, the difference was marginal with presumably negligible biological relevance (Figure 2A). However, tumoral CXCL12 stood out in this analysis, with significantly higher levels in male patients with PDA (Figure 2B). No other tumoral factor showed a differential expression by sex (Supplemental Figure S2A). Interestingly, none of the cytokines that were differentially regulated in the serum showed a difference in the tumor. To further investigate this, we performed matched serum-tumor analyses in male and female patients. We found that a significant gradient toward the serum (CXCL9, IL6, IL10, IL13) or the tumor tissue (IL1B) exists for all cytokines in question (Supplemental Figure S2B). This observation was made for male and female patients, showing similar patterns (Supplemental Figure S2B). In the analysis of the stromal compartment, no factor showed a significant difference in male and female patients (Supplemental Figure S2C).

### 3.4. Local Cytokine Changes upon Blockade of CXCL12 (Anti-CXCL12, NOX-A12) in Patients with PDA of the OPERA Trial

**Figure 3:**
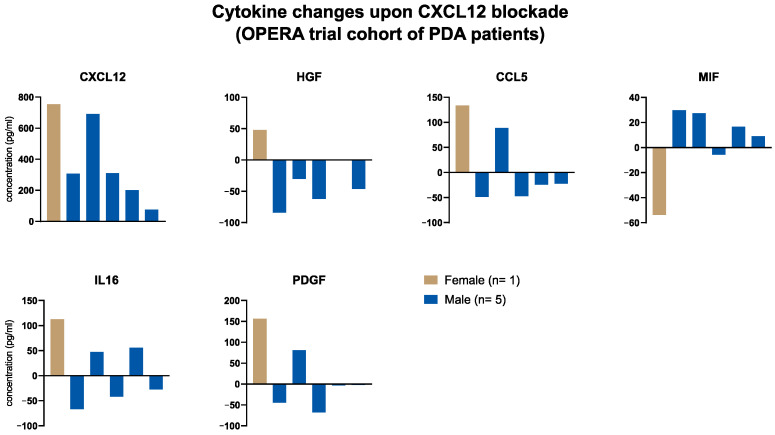
Local cytokine changes upon blockade of CXCL12 (anti-CXCL12, NOX-A12) in patients with PDA of the OPERA trial. Bar plots displaying the microenvironmental cytokine changes before (biopsy on day 0) and after (biopsy on day 14) anti-CXCL12 therapy in patients with PDA (male: *n* = 5, female: *n* = 1) of the OPERA trial cohort.

The identification of tumoral CXCL12 as a differentially regulated cytokine in the pancreatic TME indicated a sex-specific role for the targetable CXCR4-CXCL12 axis in patients with PDA. Therefore, we investigated the PDA cohort (*n* = 9) of the OPERA trial, in which patients were treated with anti-CXCL12 (NOX-A12) [17]. In six of the nine patients (one female, five males) immunological parameters from sequential biopsies before (day 0) and after (day 14) anti-CXCL12 monotherapy were available. Our aim was to assess the local effect of CXCL12 blockade and explore whether alterations in the microenvironmental immune landscape differed by sex. The results indicated that the pattern of cytokine changes upon CXCL12 blockade was different in male and female patients. On the one hand, the only female patient had the highest increase in the local concentration of CXCL12, HGF, CCL5, IL16 and PDGF (Figure 3). On the other hand, the female patient had the clearest decrease in the local MIF concentration (Figure 3). The generalizability is obviously limited, as only one female PDA patient participated in the trial. However, this fact itself underscores the importance of considering the sex of participants in the planning process of early-phase clinical trials.

### 3.5. Microenvironmental Immune Cell Infiltrate in Pancreatic Cancer Patients Stratified by Sex

**Figure 4:**
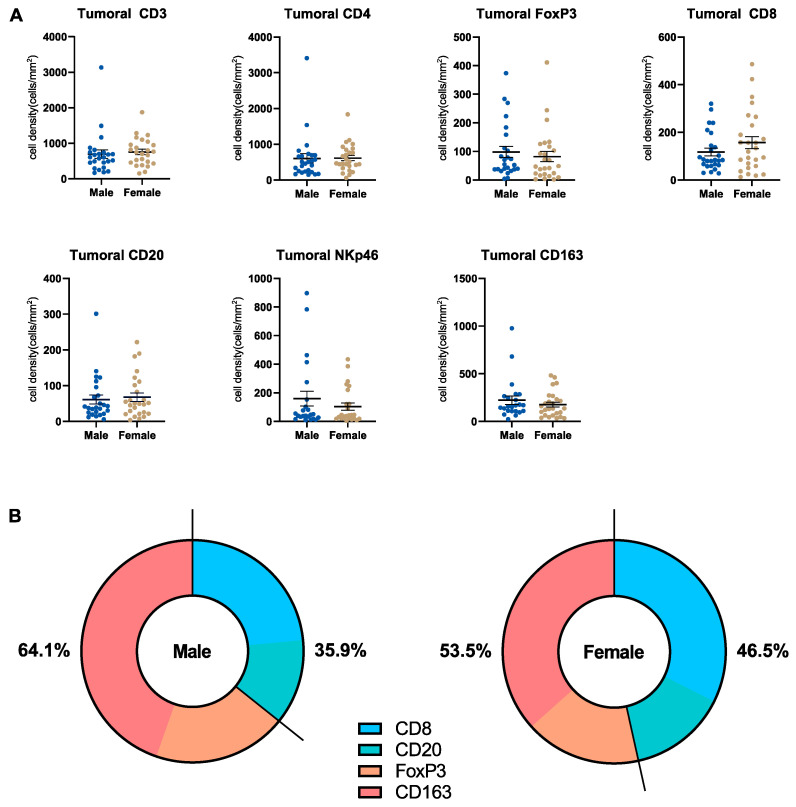
Microenvironmental immune cell infiltrate in pancreatic cancer patients stratified by sex. (**A**) Scatter dot plots comparing the tumoral densities of different immune cell types (as indicated) in male (*n* = 26) and female (*n* = 26) patients with PDA. (**B**) Pie chart comparing the ratio of (typically) pro- and anti-tumoral immune cell densities (based on mean cell density) in male (*n* = 26) and female (*n* = 26) patients with PDA.

Next, we analyzed the pancreatic immune cell infiltrate in the tumoral and stromal compartments of the TME and compared males and females with regard to this. We performed systematic immunohistochemistry (CD3, CD4, CD8, CD20, FoxP3, CD163, NKp46) and quantified the immune cells in question. Exemplary images are displayed in Supplemental Figure S3A. In the tumor compartment, we did not detect a significant difference in the number of infiltrating immune cells that was related to sex (Figure 4A). However, some interesting conclusions could be drawn. First, typically anti-tumoral immune cells (CD8^+^ and CD20^+^) showed higher numbers in females, whereas numbers of typically pro-tumoral immune cells (CD163^+^ and FoxP3^+^) were higher in males (Figure 4B). This indicates that female patients show a stronger immune control of the tumor. Secondly, a cluster of female patients with high tumoral numbers of CD8^+^ T cells (*n* = 8, with cell numbers above the mean ± SEM) was apparent. This distribution pattern of immune cells was not seen in CD3^+^ or CD4^+^ T cells. Examining the clinical characteristics of these females with high tumoral numbers of CD8^+^ T cells, some characteristics differentiated the CD8^+^ high from the CD8^+^ low group; the CD8^+^ high group had a slightly higher mean age (70.0 years vs. 66.8 years), a higher mean body weight (73.0 kg vs. 65.1 kg) and a lower rate of the tumor grade G2 (50% vs. 77.8%) (Table S2). In the stromal compartment, no relevant difference in the number of infiltrating immune cells was detected (Supplemental Figure S3B). Additionally, we analyzed CD84 gene expression using data from the TCGA pancreatic cancer via the Xena platform to evaluate the relationship between myeloid-derived suppressor cells (MDSCs) and the sex of patients with PDA [22]. CD84 gene expression did not differ by sex (Supplemental Figure S3C).

### 3.6. Survival of Patients Stratified by Sex

**Figure 5:**
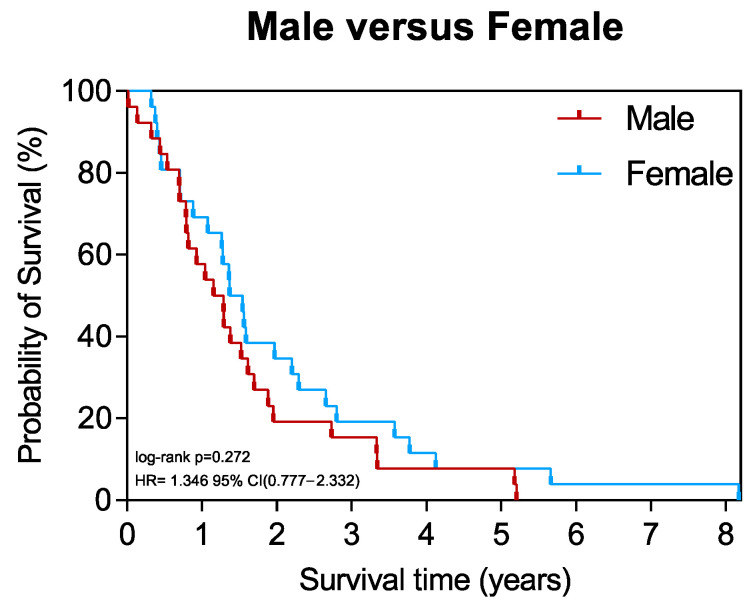
Survival of patients stratified by sex. Kaplan–Meier survival plot of male (*n* = 26) vs. female (*n* = 26) patients with PDA.

Finally, we aimed to investigate whether the overall survival differed between male and female patients with PDA. Kaplan–Meier survival analysis showed no significant difference in outcomes between males and females (Figure 5). However, females tended to have improved survival, with a mean survival of 738 days compared to 573 days in males (Table 1).

## 4. Discussion

Recent years have highlighted that many factors in malignancies vary between sexes, including innate and adaptive immunity in adult humans [7]. In health, sex differences in lymphocyte subsets, cytotoxic T cell activity, and antibody responses have been described regardless of age [7,23,24]. Testosterone has been found to have immunosuppressive effects by reducing cytokine secretion and the downregulation of Toll-like receptor 4 expression in macrophages, the inhibition of TNF synthesis and the production of anti-inflammatory IL10 [25,26,27]. In addition to myeloid cells, sex hormones also affect the functionality of antigen-presenting dendritic cells (DCs) or NK cells [28]. Specifically, estrogen (17-β-estradiol) has been shown to promote the differentiation of functional DCs from bone marrow precursor cells [29]. These effects, emphasizing the modulation of inflammatory parameters, partly explain why females are usually more susceptible to autoimmune diseases. Interestingly, estrogen also holds the potential to suppress antiviral response by DCs as observed during pregnancy [30]. With regard to tumoral immune responses, females generally mount stronger immune responses than males. In the tumor tissue, females also show features of an immune “hot” TME compared to males. However, tumor antigenicity has been reported to be lower in females [6,31]. Although these are general observations, it is important to identify immune differences by sex in specific cancer types as it is well-known that immunological features vary clearly across different types of tumors. To our knowledge, no systematic analyses of sex differences in immune responses in patients with PDA have been conducted to date.

Focusing on systemic immune features, our study found that females had higher concentrations of CXCL9, IL1B, IL6, IL10 and IL13 in their peripheral blood than males. This indicates that overall, females show signs of a stronger immune activation in PDA. CXCL9 and IL1B are two immunological serum parameters that play a controversial role in PDA. On the one hand, CXCL9 is commonly known as a chemokine with anti-tumor properties that contribute to effective T cell responses and immune infiltration, and it can serve as a favorable prognostic marker in PDA [32,33,34]. On the other hand, another study has found that CXCL9 is associated with poor patient outcomes and described it as a tumor promoter due to T cell suppression [35]. Similarly, IL1B has been correlated with increased peripheral T cell numbers and decreased TH2 cells, as well as poor overall survival in PDA [36,37]. IL6, IL10 and IL13 are normally attributed to pro-tumoral effects and poor patient outcomes [38,39,40,41]. In our cohort, a higher WBC count in females was detected which fits the stronger overall systemic immune response.

The picture of sex differences in the TME was somewhat different. None of the differentially regulated immunological factors from the peripheral blood showed a difference in the tumoral or stromal compartment between males and females. A discrepancy between local and systemic cytokine concentrations can be the result of various scenarios: The dense desmoplastic stroma in PDA represents a major barrier that can undermine effective anti-tumor responses and limit the communication between the circulation and the tumor site. Additionally, an overspill from the tumor site (tumor-driven direct dysregulation) or systemic responses as a reaction to the malignancy (tumor-driven indirect dysregulation via modulation of other organ sites or non-tumor-driven dysregulation) are potential explanations [42,43]. This phenomenon has been described in previous reports, and our findings confirm that serum findings do not automatically allow conclusions to be drawn about the local microenvironment [42,43]. The most striking finding in the TME was the higher tumoral CXCL12 concentration in males. The CXCR4-CXCL12 axis is a known immunosuppressor which leads to local T cell exclusion [44,45]. This possibly explains that females in our cohort showed a tendency towards higher numbers of anti-tumoral immune cells. Recently, an early-phase clinical study, the OPERA trial, investigated the combined inhibition of CXCL12 and PD-1 in patients with PDA demonstrating its safety [17]. We analyzed the microenvironmental changes in the PDA cohort of the trial (*n* = 6 with biopsy material before and after anti-CXCL12 therapy) and see that the only female of the patient group showed a clearly different local immune regulation. Interestingly, the female patient showed the clearest upregulation of CXCL12 levels upon CXCL12 blockade which seems counterintuitive; however, the increased levels indicate an increased binding of CXCL12 by NOX-A12 that prolongs the half-life [17]. As a speculation, the higher turnover of CXCL12 production could lead to this massive increase in bound CXCL12 within the tissue. This also underlines the complexities; apart from concentration, one can also consider the production/degradation of cytokines as a relevant factor underlying sex differences. Despite the low number of cases explored, our findings implicate that immunotherapeutic approaches may have different effects by sex. Surely, these findings are of limited generalizability because only one of six patients was female. However, this underlines that preclinical and clinical studies need to be focused on detecting sex differences, as demanded by the sex and gender equity in research (SAGER) guidelines [46]. With all the evidence in hand, including our study, it would be wrong to assume that findings from males apply automatically to females. This implies that for specific interventions such as the targeting of CXCL12, it is reasonable to explore sex-specific expression and turnover differences for stratification of clinical trial participants.

Finally, the investigation of the clinical aspect with overall survival did not show a significant difference between the sexes. However, females tended towards improved outcomes with a higher mean survival (738 days vs. 573 days). This is in line with prior findings [1,2,12].

The limitations of this work are the rather small cohorts (only one female in the OPERA trial analysis) and the large complexity (hormonal status, dynamics of endocrine biology in aging patients, hormone-like effects of concomitant drugs, KRAS mutation status, etc.). However, on the other hand, the presented cohorts are reflective of the common situation in early-phase clinical trials, which are decisive for further clinical (drug) development.

## 5. Conclusions

The results of our study show for the first time that systemic and local immune responses in PDA can differ between males and females. This underscores that sex-optimized medicine is a clinical need and should be considered specifically in pancreatic cancer research. For specific immunological interventions ranging from cellular therapies to targeted immunomodulation, it is advisable to specifically investigate sex-dependent differences before clinical translation.

## Figures and Tables

**Table 1:** Patient characteristics.

		Male	Female	Total	*p*-Value
Characteristic		(*n* = 26)	(*n* = 26)	(*n* = 52)	
Age (years)		64.5 ± 1.8	66.5 ± 2.2	65.5 ± 1.4	-
>/≤Average age (*n*/*n*)		13/13	16/10	-	0.412
Body weight (kg)		75.4 ± 2.4	66.6 ± 2.1	71.5 ± 1.8	-
>/≤Body weight (*n*/*n*)		13/13	5/21	-	0.019
BMI		23.9 ± 2.3	25.2 ± 3.0	24.5 ± 1.8	-
>/≤BMI (*n*/*n*)		7/19	9/17	-	0.557
Diabetes mellitus (*n*)		7	8	15	0.765
Glucocorticoid or immunosuppressive drug intake (*n*)		1	0	1	0.322
Cardiovascular comorbidities (*n*)		10	17	27	0.053
Pulmonary comorbidities (*n*)		2	3	5	0.646
Renal comorbidities (*n*)		1	2	3	0.561
Hepatic comorbidities (*n*)		0	0	0	n.a.
Autoimmune comorbidities (*n*)		1	1	2	>0.999
T	T3 (*n*)	25	26	51	0.322
	T4 (*n*)	1	0	1	
N	N0 (*n*)	4	5	9	0.491
	N1 (*n*)	22	21	43	
M	M0 (*n*)	23	25	48	0.307
	M1 (*n*)	3	1	4	
Grade	G2 (*n*)	15	18	33	0.397
	G3 (*n*)	11	8	19	
R ^a^	R0 (*n*)	4	2	6	0.367
	R1 (*n*)	21	24	45	
Survival days		573 ± 98	738 ± 131	655 ± 82	0.619
>/≤ Survival days (*n*/*n*)		7/19	9/17	-	

Data are shown as mean ± SEM. BMI = body mass index (calculated as weight in kilograms divided by height in meters squared), n.a. = not applicable, T = stage of primary tumor, *n* = regional lymph node status, M = distant metastasis status, R = resection margin status. ^a^ Information available on resection margin status from 51 patients.

## Data Availability

All data are available upon request. All data relevant to the study are included in the article.

## Supplementary Materials

The following supporting information can be downloaded at: https://www.mdpi.com/article/10.3390/cancers15061815/s1, Figure S1: Female pancreatic cancer patients show a stronger systemic immune response; Figure S2: Male patients express higher tumoral levels of CXCL12; Figure S3: Microenvironmental immune cell infiltrate in pancreatic cancer patients by sex; Table S1: Female patient characteristics (IL13 high vs. IL13 low subgroup); Table S2: Female patient characteristics (CD8 high vs. CD8 low subgroup).
