# Supplementary material for: Sex Differences in the Systemic and Local Immune Response of Pancreatic Cancer Patients
Source: Cancers (Basel). 2023 Mar 17;15(6):1815. doi: 10.3390/cancers15061815 (PMC10047039; doi:10.3390/cancers15061815)
Supplement: Supplementary file 1 [file cancers-15-01815-s001.zip › cancers-2184475-supplementary.pdf]

SUPPLEMENTAL MATERIAL

Sex differences in the systemic and local immune response of pancreatic cancer patients

Azaz Ahmed[1,2], Sophia Köhler[1], Rosa Klotz[3], Nathalia Giese[3], Thilo Hackert[3], Christoph Springfeld[1], Dirk Jäger[1,4], Niels Halama[1,2]

[1] Medical Oncology and Internal Medicine VI, National Center for Tumor Diseases (NCT), University Hospital Heidelberg, University Heidelberg, 69120 Heidelberg, Germany
[2] Translational Immunotherapy, German Cancer Research Center (DKFZ), 69120 Heidelberg, Germany
[3] General, Visceral and Transplantation Surgery, University Hospital Heidelberg, University Heidelberg, 69120 Heidelberg, Germany
[4] Applied Tumor Immunity Clinical Cooperation Unit, National Center for Tumor Diseases (NCT), German Cancer Research Center (DKFZ), 69120 Heidelberg, Germany

Supplemental Figure S1

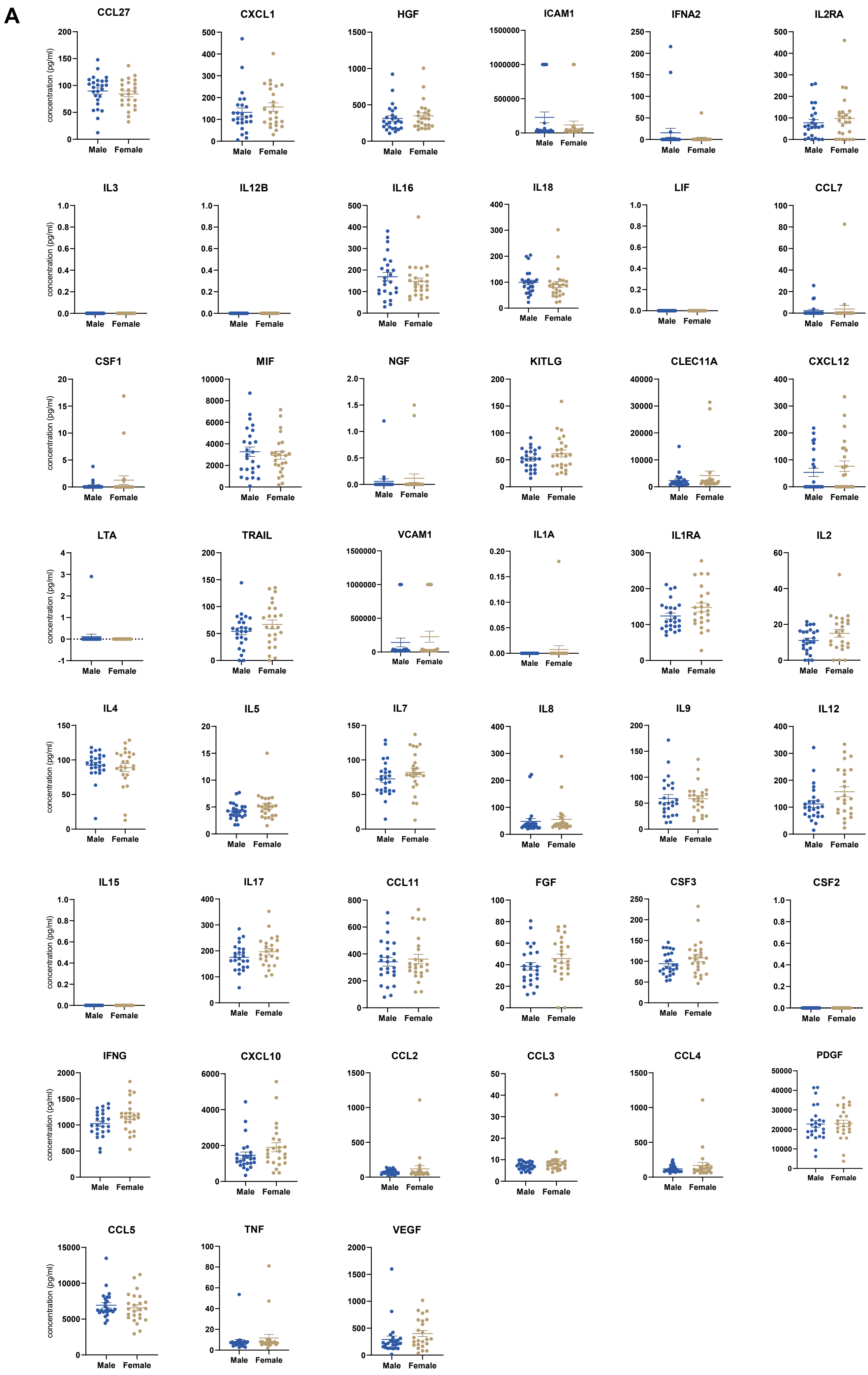

Supplemental Figure S1

B **Correlation of serum IL6 and CRP levels**

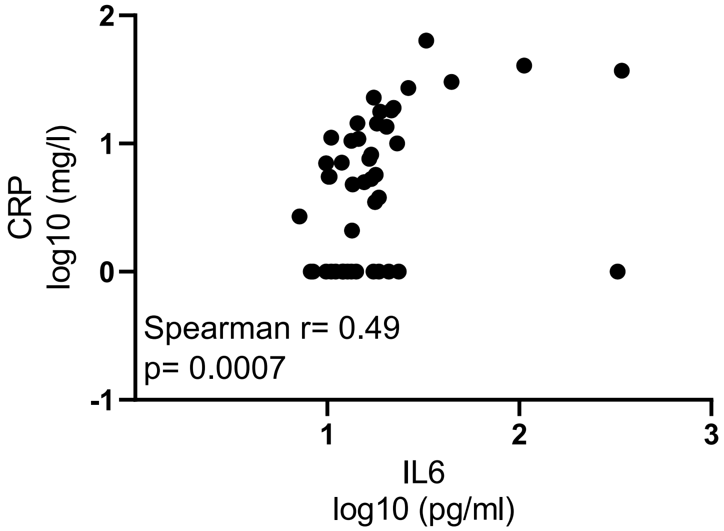

Supplemental Figure S1

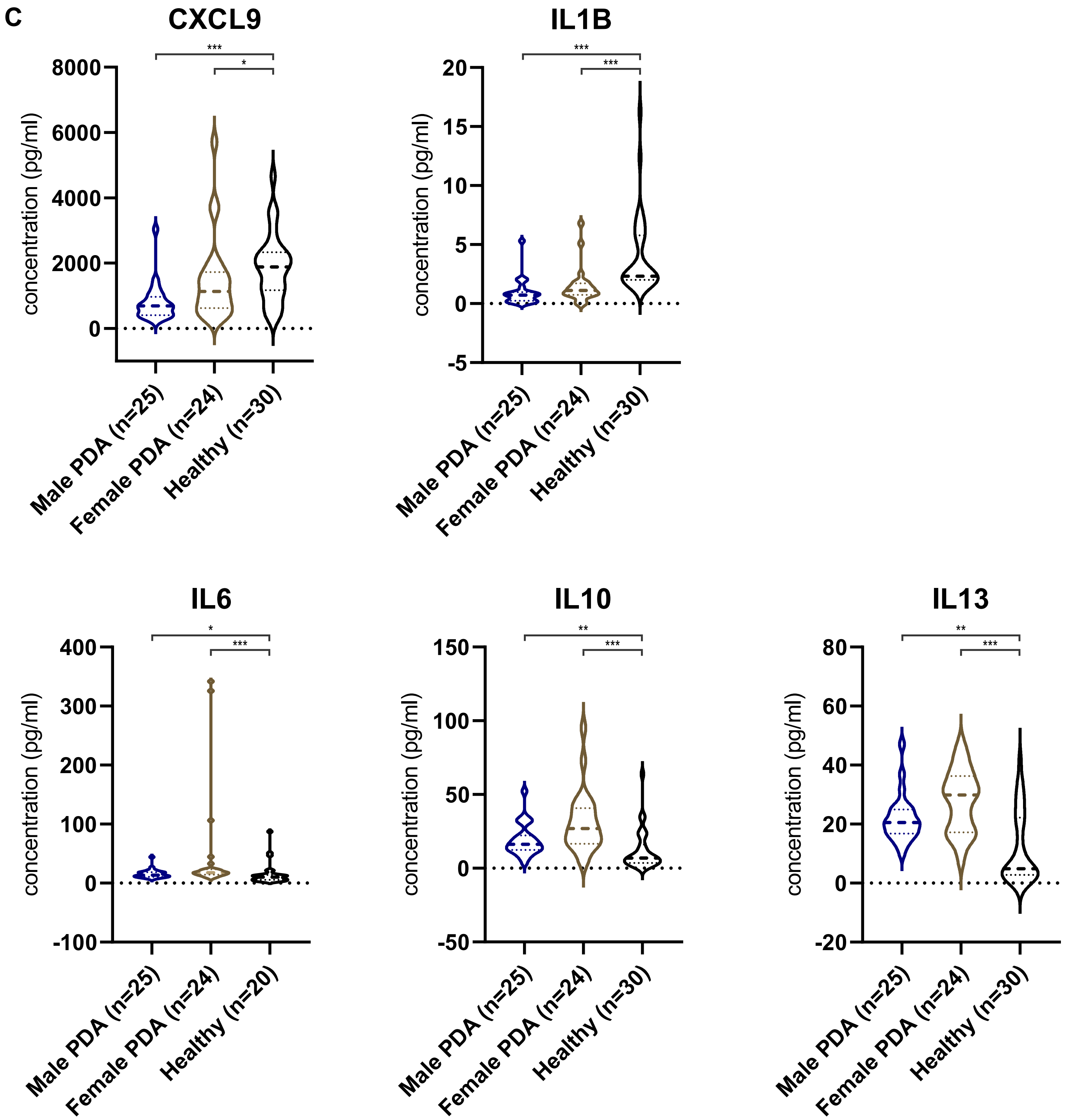

Supplemental Figure S2

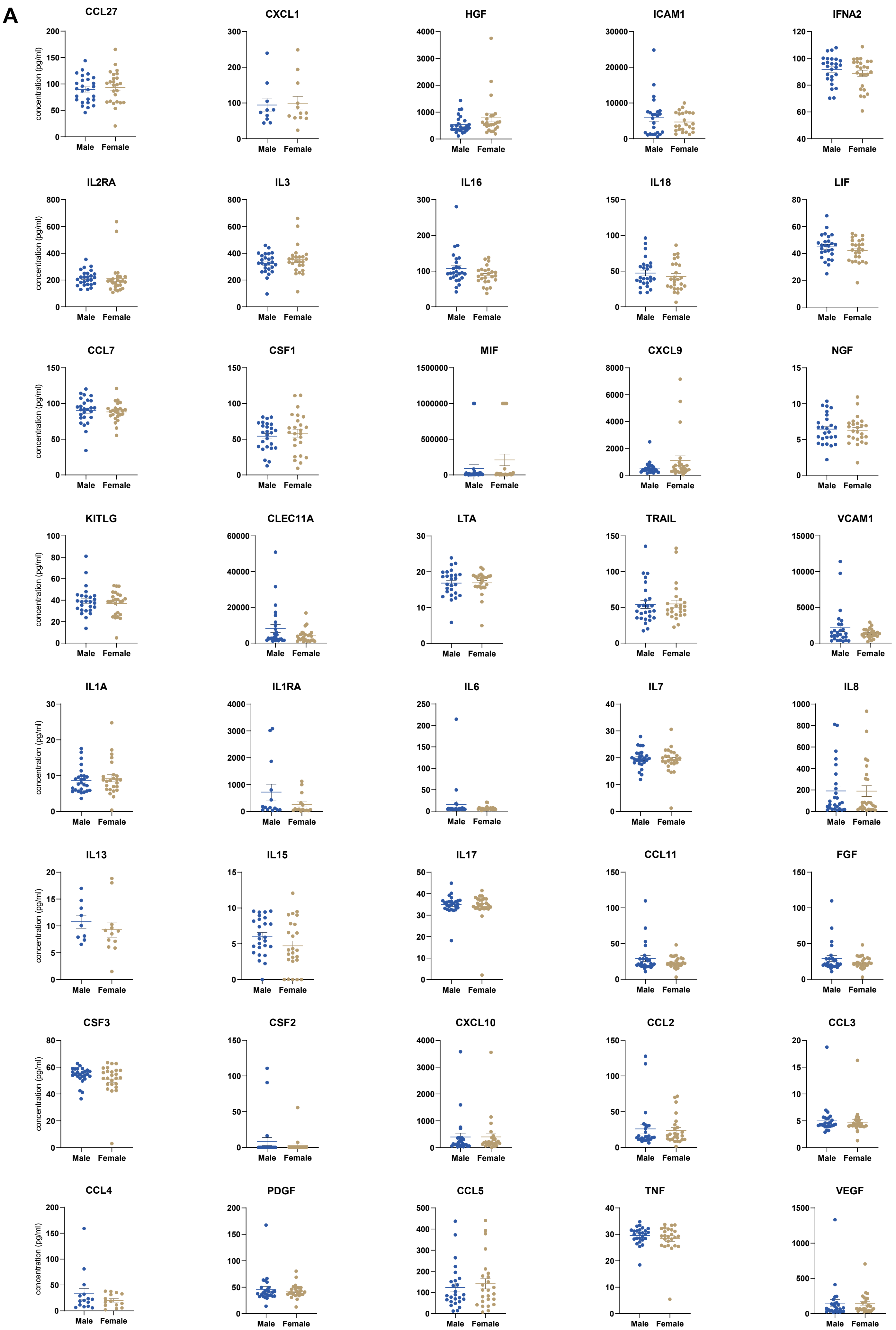

Supplemental Figure S2

B

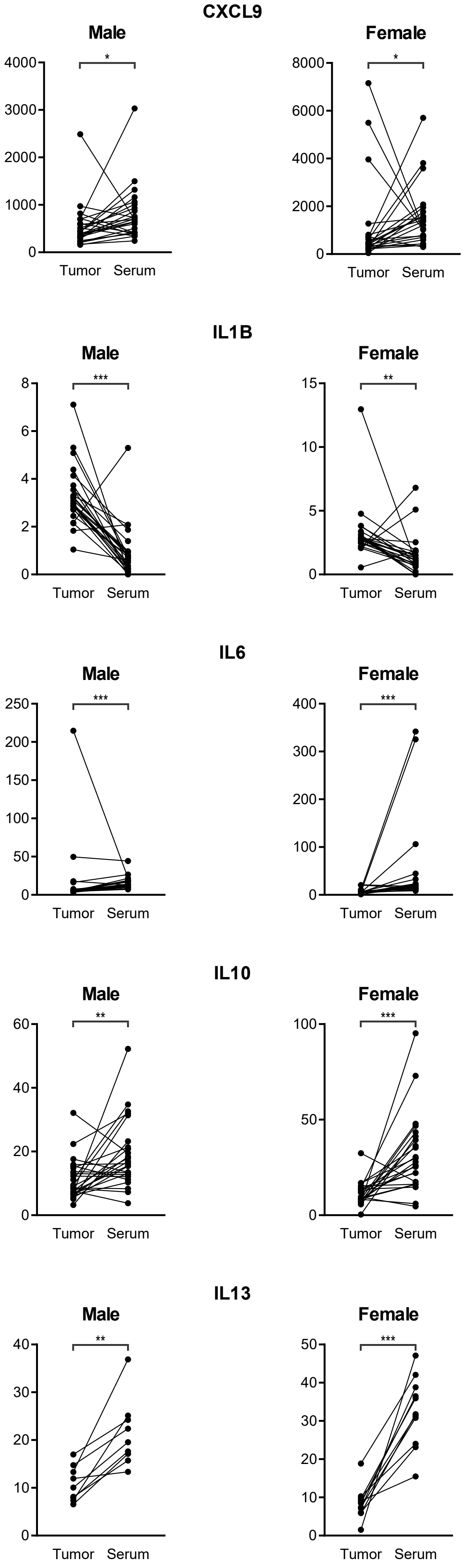

Supplemental Figure S2

C

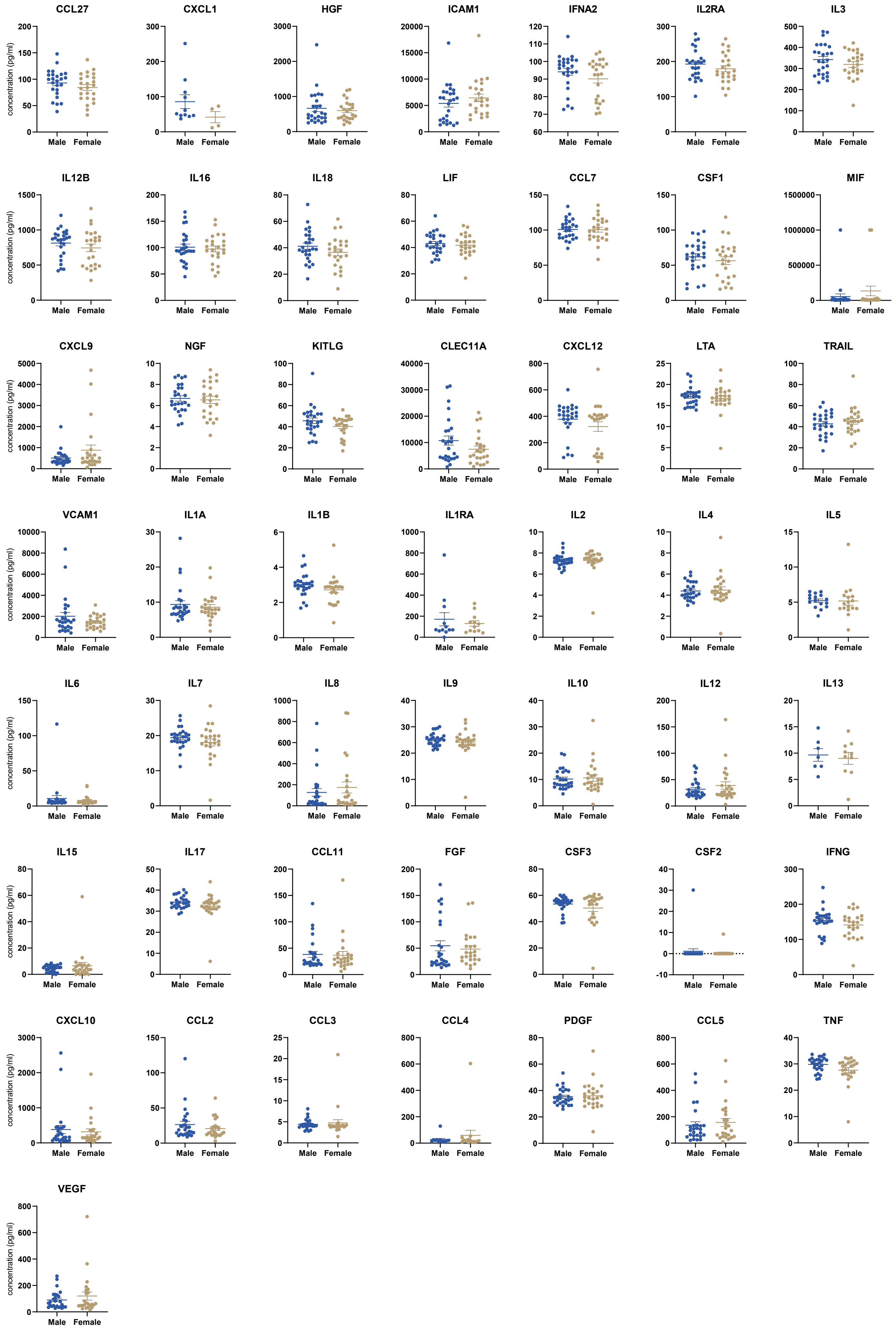

Supplemental Figure S3

A

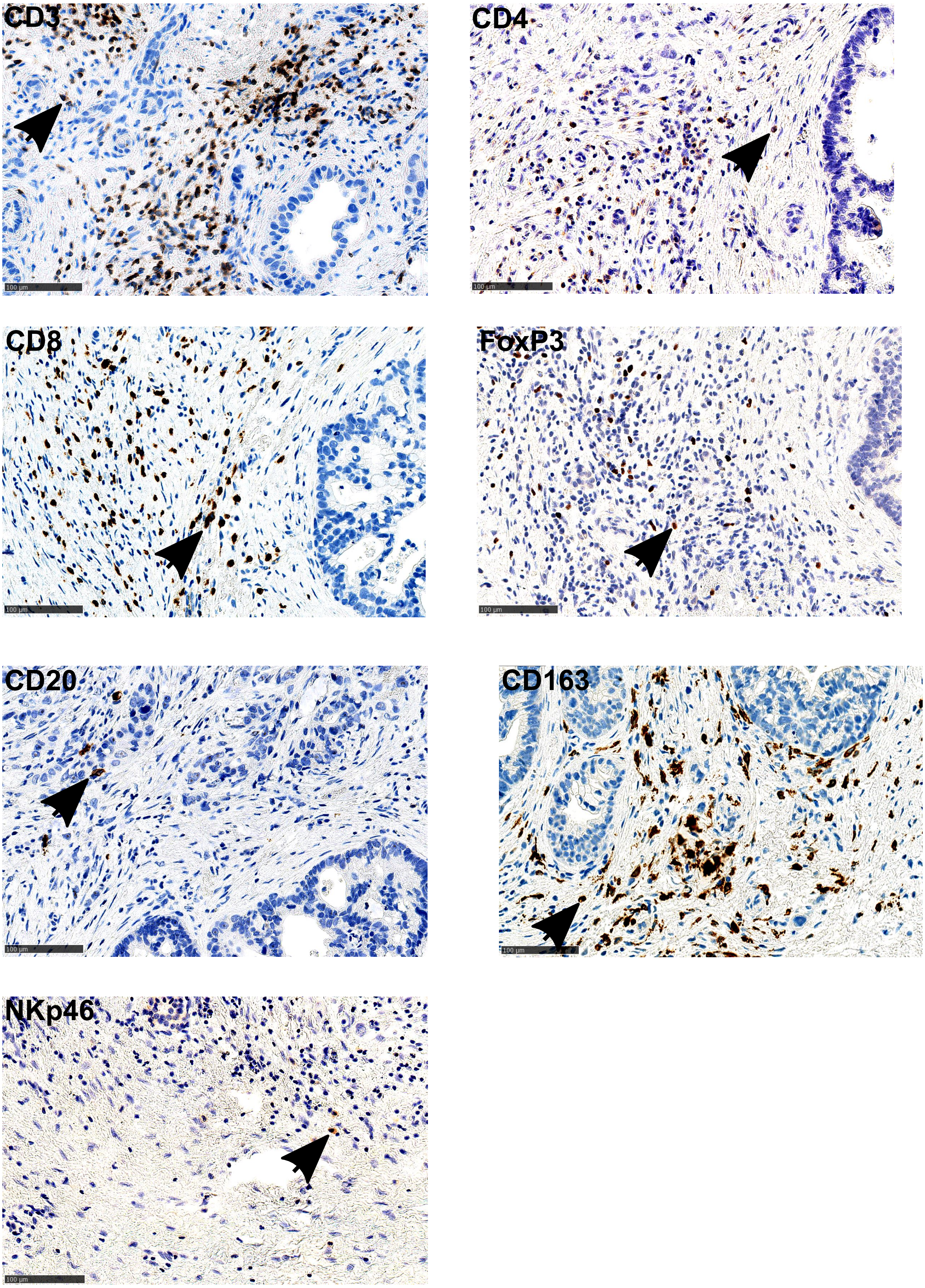

Supplemental Figure S3

B

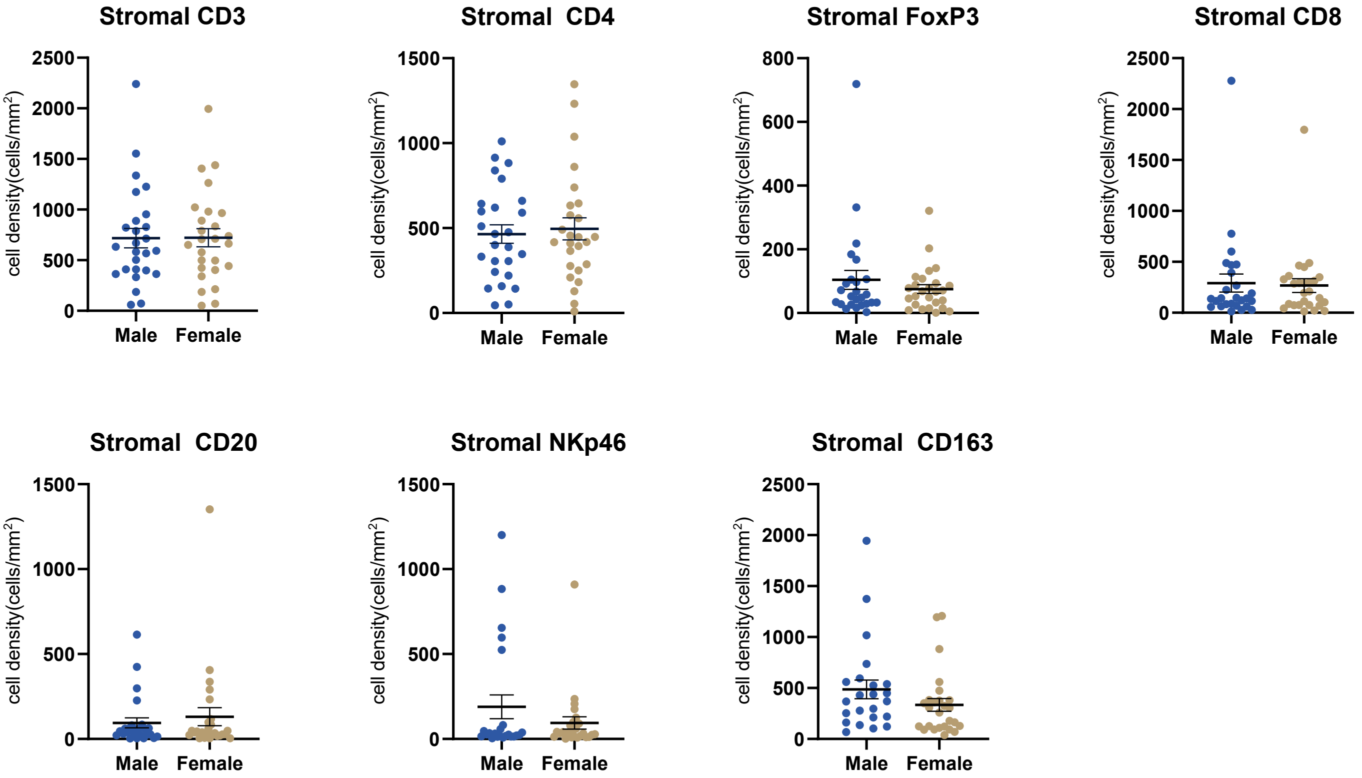

C

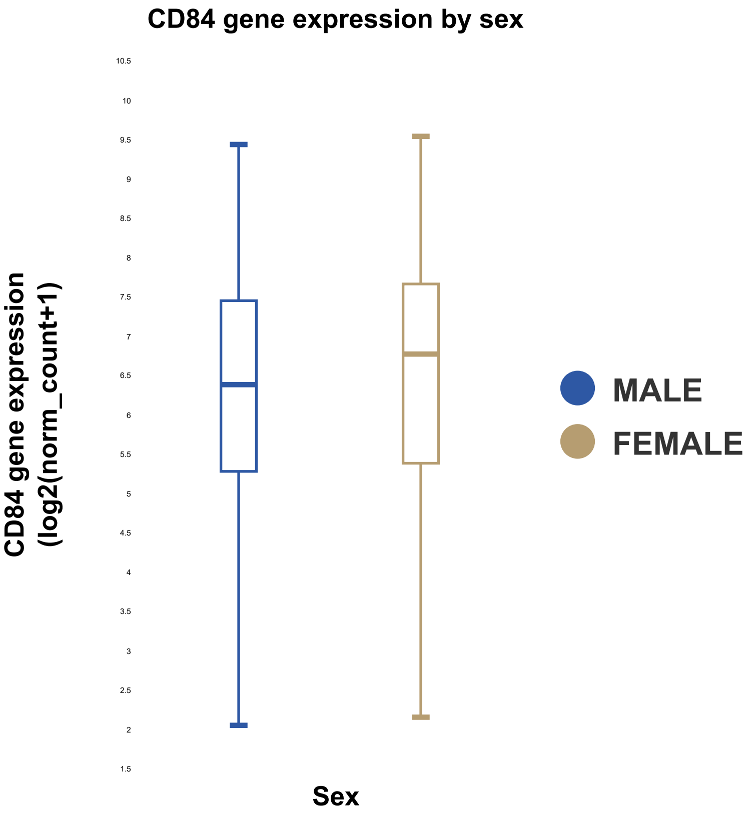

SUPPLEMENTAL FIGURE LEGENDS

Supplemental Figure S1 Female pancreatic cancer patients show a stronger systemic immune response

(A) Scatter dot plots comparing the serum concentration of immunological parameters (as indicated) in male (n= 25) and female (n= 24) PDA patients.

(B) Correlation analysis of systemic IL6 and CRP levels in all patients (n= 48).

(C) Violin plots comparing serum concentration of immunological parameters (as indicated) in healthy individuals (n=30) versus male (n=25) and female (n=24) PDA patients.

PDA= Pancreatic ductal adenocarcinoma. CRP= C-reactive protein. *$p \leq 0.05$, **$p \leq 0.005$, ***$p \leq 0.0001$.

Supplemental Figure S2 Male patients express higher tumoral levels of CXCL12

(A) Scatter dot plots comparing the tumoral concentration of immunological parameters (as indicated) in male (n= 26) and female (n= 26) PDA patients.

(B) Comparison of male (n=25) and female (n=24) patients regarding the serum-to-tumor gradient of immunological parameters (as indicated).

(C) Scatter dot plots comparing the stromal concentration of immunological parameters (as indicated) in male (n= 26) and female (n= 24) PDA patients.

PDA= Pancreatic ductal adenocarcinoma. *$p \leq 0.05$, **$p \leq 0.005$, ***$p \leq 0.0001$.

Supplemental Figure S3 Microenvironmental immune cell infiltrate in pancreatic cancer patients by sex

(A) Immunohistochemistry for immune cell types (as indicated). Examples of positively stained cells are highlighted (arrows). Scale bars, 100µm.

(B) Scatter dot plots comparing the stromal densities of different immune cell types (as indicated) in male (n= 26) and female (n= 26) PDA patients.

(C) Box plot comparing CD84 gene expression in male (n= 101) and female (n= 82) PDA patients. The data was obtained from the Xena platform.

PDA= Pancreatic ductal adenocarcinoma.

SUPPLEMENTAL TABLES

Supplemental Table S1. Female patient characteristics (IL13 high vs. IL13 low subgroup)

		Female		Total
**Characteristic**		**IL13 high subgroup (n= 12)**	**IL13 low subgroup (n= 14)**	**(n= 26)**
**Age (years)**		69.9 ± 3.6	63.6 ± 2.6	66.5 ± 2.2
**Body weight (kg)**		68.7 ± 3.9	64.9 ± 2.2	66.6 ± 2.1
**BMI**		26.5 ± 5.0	24.2 ± 3.7	25.2 ± 3.0
**Diabetes mellitus (n)**		5	3	8
**Immunosuppressive drug intake (n)**		0	0	0
**Cardiovascular comorbidities (n)**		10	8	18
**Pulmonary comorbidities (n)**		2	1	3
**Renal comorbidities (n)**		0	2	2
**Hepatic comorbidities (n)**		0	0	0
**Autoimmune comorbidities (n)**		1	0	1
**T**	T3 (n)	12	14	26
	T4 (n)	0	0	0
**N**	N0 (n)	3	2	5
	N1 (n)	9	12	21
**M**	M0 (n)	11	14	25
	M1 (n)	1	0	1
**Grade**	G2 (n)	8	10	18
	G3 (n)	4	4	8
**R**	R0 (n)	2	0	2
	R1 (n)	10	14	24

Data are shown as mean ± SEM. BMI= body mass index (calculated as weight in kilograms divided by height in meters squared), T= stage of primary tumor, N= regional lymph node status, M= distant metastasis status, R= resection margin status.

Supplemental Table S2. Female patient characteristics (CD8 high vs. CD8 low subgroup)

		Female		Total
**Characteristic**		**CD8 high subgroup (n= 8)**	**CD8 low subgroup (n= 18)**	**(n= 26)**
**Age (years)**		70.0 ± 5.9	66.8 ± 2.6	66.5 ± 2.2
**Body weight (kg)**		73.0 ± 8.0	65.1 ± 1.7	66.6 ± 2.1
**BMI**		27.5 ± 8.8	24.7 ± 2.9	25.2 ± 3.0
**Diabetes mellitus (n)**		2	6	8
**Immunosuppressive drug intake (n)**		0	0	0
**Cardiovascular comorbidities (n)**		6	12	18
**Pulmonary comorbidities (n)**		0	3	3
**Renal comorbidities (n)**		0	2	2
**Hepatic comorbidities (n)**		0	0	0
**Autoimmune comorbidities (n)**		0	1	1
**T**	T3 (n)	8	18	26
	T4 (n)	0	0	0
**N**	N0 (n)	1	4	5
	N1 (n)	7	14	21
**M**	M0 (n)	8	17	25
	M1 (n)	0	1	1
**Grade**	G2 (n)	4	14	18
	G3 (n)	4	4	8
**R**	R0 (n)	0	2	2
	R1 (n)	8	16	24

Data are shown as mean ± SEM. BMI= body mass index (calculated as weight in kilograms divided by height in meters squared), T= stage of primary tumor, N= regional lymph node status, M= distant metastasis status, R= resection margin status.
